# Mitochondrial genome of the garfish *Hyporhamphus quoyi* (Beloniformes: Hemiramphidae) and phylogenetic relationships within Beloniformes based on whole mitogenomes

**DOI:** 10.1371/journal.pone.0205025

**Published:** 2018-11-15

**Authors:** Lei Cui, Yuelei Dong, Rongbo Cao, Jian Gao, Jingyi Cen, Zhijia Zheng, Songhui Lu

**Affiliations:** Key Laboratory of Eutrophication and Red Tide Prevention, Research Center for Harmful Algae and Marine Biology, Jinan University, Guangzhou, China; National Cheng Kung University, TAIWAN

## Abstract

Mitochondrial DNA (mtDNA) can provide genome-level information (e.g. mitochondrial genome structure, phylogenetic relationships and codon usage) for analyzing molecular phylogeny and evolution of teleostean species. The species in the order Beloniformes have commercial importance in recreational fisheries. In order to further clarify the phylogenetic relationship of these important species, we determined the complete mitochondrial genome (mitogenome) of garfish *Hyporhamphus quoyi* of Hemiramphidae within Beloniformes. The mitogenome was 16,524 bp long and was typical of other teleosts mitogenomes in size and content. Thirteen PCGs started with the typical ATG codon (with exception of the cytochrome coxidase subunit 1 (*cox1*) gene with GTG). All tRNA sequences could be folded into expected cloverleaf secondary structures except for tRNA^Ser (AGN)^ which lost a dihydrouracil (DHU) stem. The control region was 866 bp in length, which contained some conserved sequence blocks (CSBs) common to Beloniformes. The phylogenetic relationship between 26 fish Beloniformes species was analyzed based on the complete nucleotide and amino acid sequences of 13 PCGs by two different inference methods (Maximum Likelihood and Bayesian Inference). Phylogenetic analyses revealed Hemiramphidae as the sister group to Exocoetidae and it is a paraphyletic grouping. Our results may provide useful information on mitogenome evolution of teleostean species.

## 1. Introduction

Mitochondrial DNA (mtDNA) of teleosts is a circular genome ranging from 15 to 19 kbp in length that is generally composed of two ribosomal RNA genes (12S rRNA and 16S rRNA), 13 protein-coding genes (PCGs), 22 transfer RNA genes (tRNAs) and two typical non-coding control regions (origin of the light strand (O_L_) and control region (CR)) which contain essential regulatory elements for replication and transcription [1, 2]. MtDNA is commonly used for population genetics and phylogenetic molecular evolution due to maternal inheritance, rapid evolution, coding content conservation, and high substitution rates compared to the nuclear genome[3, 4]. In addition, the molecular characteristics of the mitogenome, such as gene rearrangement, tRNA secondary structure and models of the control of mtDNA replication are valuable for deep phylogenetic analysis [5, 6].

Garfishes (order Beloniformes), which are known for their importance to commercial and recreational fisheries, consist of approximately 260 species classified into 6 families depending on the taxonomy [7]. Identifying adult garfish is not difficult [7], but larvae identification is difficult to carry out based on morphological characters. Several partial mitochondrial CRs gene sequences from Beloniformes have been sequenced and used for systematics [8]. However, the CRs do not provide enough phylogenetic information for molecular evolution and sometimes even appear of disputation. Although other researchers had previously determined the complete mitogenomes of some species from Beloniformes and constructed a phylogenetic tree to analyse their interspecies relationship[2], we still do not understand the higher-level phylogeny of Beloniformes because of the lack of more completely sequenced mitogenomes that will allow obtaining more informationfor a deeper exploration and evolutionary relationships. So far, there are 35 described variations that deviated from conserved mtDNA organization in teleosts, although none described among Beloniformes[9]. Therefore, sequencing more Beloniformes mtDNA may show novel variations in mtDNA organization among vertebrates. To date, more than 200 complete mitogenomes have been determined from teleostean species, however, only 26 species from Beloniformes are available in the GenBank database. The garfish *Hyporhamphus quoyi*, which is zooplankton feeders and carnivores[10], is a widespread species in the family Hemiramphidae (Beloniformes) ranging from Southeast Asia, Oceania, the eastern Pacific Ocean and West Africa[7]. At present, the complete mitogenome of *H*. *quoyi* has not been sequenced. To understand the deeper interspecies relationships of Beloniformes, we sequenced the complete mitochondrial genome of *H*. *quoyi* and its genome organization and structure were compared with other Beloniformes fish. In addition, the phylogenetic tree has been reconstructed by the Bayesian inference (BI) and Maximum Likelihood (ML) methods to understand the evolutionary relationships among Beloniformes. The characterization of the *H*. *quoyi* mitogenome may provide more information about the evolution of teleosts and will aid in larvae identifications.

## 2. Methods

### Sample collection, DNA extraction, PCR amplification and sequencing

Adult specimens of *H*. *quoyi* were collected in the Pearl River estuary (N 21°45′, E 133°36′), China, in June 2017 and no specific permissions were required for this location. According to the International Union for Conservation of Nature Red List, *H*. *quoyi* were not protected or endangered species. Our study was conducted with the approval from the Institutional Animal Care and Use Committee at Jinan University. All operations were performed according to international guidelines concerning the care and treatment of experimental animals. All samples were preserved in 95% ethanol and were stored at -80°C until use. Total genomic DNA was isolated from dorsal muscle tissue samples using proteinase K treatment, followed by the Animal Tissue Genomic DNA Extraction Kit. To sequence the *H*. *quoyi* mitogenome, several primer pairs were designed for the amplification according to the conservative sequence based on the conserved sequences which were obtained by aligning the complete mitogenome of *Hyporhamphus sajori* (GenBank: AB370892.1) and *Hyporhamphus intermedius*(GenBank: NC_026467.1) (S1 Table)[2]. PCR amplification reactions were performed with PrimeSTAR^®^ GXL DNA Polymerase under the following conditions: after an initial denaturation step at 95°C for 1 min, then 35 cycles at 95°C for 20 s (denaturation), 55°C for 45 s (annealing) and 72°C for 1–5 min (elongation). PCR products were sequenced from both directions using a primer walking method.

### Sequence annotation and analysis

We used the program Seqman within Lasergene software to check and assemble manually the mitogenome sequences of *H*. *quoyi*. The complete sequence and its annotation were performed by NCBI BLAST (http://blast.ncbi.nlm.nih.gov/Blast) and the DNAStar package (DNAStar Inc. Madison, WI, USA). The circular gene map of mitogenome was drawn by GCView Server[11]. The location of the 13 PCGs and the two rRNAs were primarily determined through Dual Organellar Genome Annotator (DOGMA)[12]. All of the tRNA gene sequences were identified by the tRNA-scan-SE1.21 from the website http://lowelab.ucsc.edu/tRNAscan-SE/ using the default search mode and the ‘Mito/chloroplast’ source[13]. The software RNAstructure was used to draw the secondary structure of tRNA genes and O_L_[14]. The relative synonymous codon usage (RSCU) of the 13 PCGs was calculated by the software MEGA 6[15]. Tandem repeats in the control region (CR) were analysed using the Tandem Repeats Finder program (http://tandem.bu.edu/trf/trf.html)[16]. The nucleotide composition skewness was measured according to the following formulas: AT skew [(A−T)/(A+T)] and GC skew [(G−C)/(G+C)][17]. To analyse evolutionary adaptation, the rates of nonsynonymous (*Ka*) and synonymous (*Ks*) substitutions in the mtDNA among 26 garfish of Beloniformes were estimated with DnaSP 5.10.01 [18]. The complete mitochondrial DNA sequence of the *H*. *quoyi* was deposited into the GenBank database under the accession number MG851912.1.

### Phylogenetic analysis

A total of 26 Beloniformes mitogenomes available in GenBank were used to investigate the phylogenetic relationships among fish (Table 1). The mitogenome of Perciformes fish(*Caesio cuning* (KP874185.1), *Emmelichthys struhsakeri* (AP004446.1) and *Banjos banjos* (KT345965.1)) was used as outgroups[19–21]. The nucleotide and amino acid sequences of the 13 PCGs were aligned using default settings and concatenated, which were used for phylogenetic analysis via BI and ML methods by MrBayes v 3.2.4 and raxmlGUI, respectively[22, 23]. Each gene was aligned separately by the software Clustal X with default settings[24]. GTR+ I+ G was selected as the appropriate model for the nucleotide sequences by Modeltest 3.7 based on Akaike’s information criterion (AIC)[25]. MtArt+ I+ G+ F was the appropriate model for the amino acid sequence dataset according to ProtTest 3.4 based on AIC[26]. For the Bayesian Inference, four independent runs were allowed to run simultaneously for 1,000,000 generations and each was sampled every 1,000 generations, with the first 25% discarded as burn-in. Stationarity was considered to be reached when the average standard deviation of split frequencies was much less than 0.01. In ML analysis, the default parameters were used and the node support values were assessed by bootstrap resampling (BP) estimated using 100 replicates. The resulting phylogenetic trees were drawn by FigTree v1.4.3.

**Table 1:** Summary of the base composition of the mitogenomes at each codon position of the concatenated the 13 PCGs across 27 Beloniformes species.

Family	Species	Accession number	Size (bp)	Whole genome composition							PCGs	
				A%	G%	T%	C%	A+T%	AT skew	GC skew	AT skew	GC skew
Adrianichthyidae	*Oryzias curvinotus*	NC_034775.1	16676	27.72	17.40	26.48	28.41	56.13	-0.0124	-0.2069	-0.1015	-0.2350
Adrianichthyidae	*Oryzias dancena*	GU013789.1	16863	29.00	16.52	23.85	30.63	59.63	-0.0272	-0.1814	-0.1194	-0.1984
Adrianichthyidae	*Oryzias javanicus*	GU013790.1	16890	26.77	17.78	28.12	27.32	54.10	-0.0102	-0.2253	-0.1127	-0.2512
Adrianichthyidae	*Oryzias latipes*	NC_004387.1	16714	27.26	17.95	26.52	28.28	55.53	-0.0183	-0.1927	-0.1156	-0.2264
Adrianichthyidae	*Oryzias luzonensis*	NC_012979.1	16666	26.42	18.62	27.63	27.34	53.76	-0.0171	-0.1948	-0.1154	-0.2155
Adrianichthyidae	*Oryzias melastigma*	NC_018546.1	16864	28.94	16.53	23.82	30.71	59.65	-0.0297	-0.1806	-0.1210	-0.1954
Adrianichthyidae	*Oryzias minutillus*	NC_012975.1	16953	29.31	17.31	24.01	29.36	58.67	-0.0009	-0.1621	-0.0986	-0.1756
Adrianichthyidae	*Oryzias sarasinorum*	AB370891.1	16462	29.18	17.16	24.93	28.73	57.91	0.0339	-0.2740	-0.0900	-0.2031
Adrianichthyidae	*Oryzias sinensis*	NC_013434.1	16654	29.21	16.37	26.57	27.85	57.05	0.0077	0.1846	0.0900	0.2031
Adrianichthyidae	*Xenopoecilus sarasinorum*	NC_011172.1	16462	29.18	17.16	24.93	28.73	57.91	0.0077	-0.1846	-0.0900	-0.2031
Belonidae	*Strongylura anastomella*	NC_026998.1	16654	29.21	16.37	26.57	27.85	57.05	0.0077	0.1846	0.0900	0.2031
Belonidae	*Tylosurus acus*	KU605633.1	16723	29.03	17.13	26.22	27.62	56.65	0.0249	-0.2097	-0.0456	-0.2220
Belonidae	*Ablennes hians*	NC_011180.1	16825	30.00	14.65	27.09	28.26	58.26	0.0298	-0.2980	-0.0496	-0.3447
Exocoetidae	*Parexocoetus brachypterus*	NC_036719.1	16776	29.05	15.90	27.91	27.14	56.19	0.0339	0.2740	-0.0513	-0.3113
Exocoetidae	*Prognichthys sealei*	NC_036722.1	16527	27.80	17.46	26.17	28.58	56.38	-0.0137	-0.1997	-0.0612	-0.2717
Exocoetidae	*Cheilopogon agoo*	NC_036720.1	16526	29.47	16.10	26.92	27.51	56.98	0.0345	-0.2514	-0.0493	-0.2885
Exocoetidae	*Cheilopogon atrisignis*	NC_029730.1	16530	28.77	16.64	27.34	27.24	56.01	0.0273	-0.2433	-0.0553	-0.2770
Exocoetidae	*Cheilopogon cyanopterus*	NC_036721.1	16529	28.92	16.49	27.35	27.24	56.16	0.0298	-0.2476	-0.0536	-0.2813
Exocoetidae	*Cheilopogon doederleinii*	NC_033541.1	16525	29.23	16.30	27.16	27.32	56.54	0.0338	-0.2500	-0.0495	-0.2820
Exocoetidae	*Cheilopogon unicolor*	NC_029728.1	16529	29.06	16.40	27.24	27.31	56.37	0.0310	-0.2485	-0.0511	-0.2813
Exocoetidae	*Cypselurus hiraii*	NC_007403.1	16528	29.91	15.63	26.83	27.56	57.47	0.0409	-0.2638	-0.0360	-0.3034
Exocoetidae	*Exocoetus volitans*	NC_003184.1	16527	28.35	17.12	27.19	27.34	55.69	0.0180	-0.2273	-0.0658	-0.2594
Hemiramphidae	*Hyporhamphus intermedius*	NC_026467.1	16720	26.71	16.59	27.08	29.62	56.33	-0.0517	-0.2401	-0.1519	-0.2652
Hemiramphidae	*Hyporhamphus sajori*	AB370892.1	16721	26.69	16.61	27.84	28.86	55.55	-0.0391	-0.2527	-0.1396	-0.2802
Hemiramphidae	*Hyporhamphus quoyi*	MG851912.1	16525	29.28	15.33	27.86	27.53	56.80	0.0307	-0.2899	-0.0598	-0.3195
Scomberesocidae	*Cololabis saira*	NC_003183.1	16499	30.42	14.76	25.73	29.09	59.51	0.0224	-0.2711	-0.0704	-0.2986
Zenarchopteridae	*Dermogenys pusilla*	NC_034337.1	16529	30.70	14.60	26.50	28.19	58.89	0.0425	-0.2895	-0.0442	-0.3340

## Results and discussion

### Genome organization and structure

**Fig 1:**
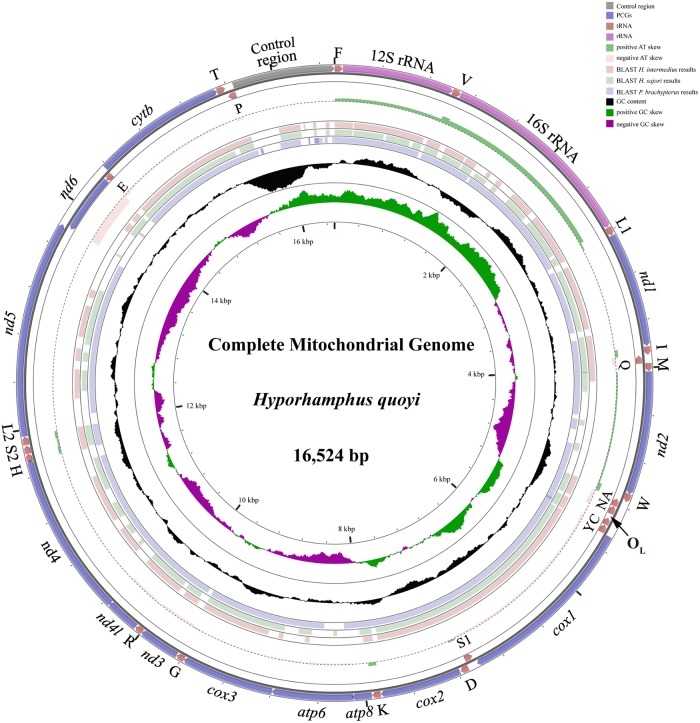
Circular map of the mitogenome of *Hyporhamphus quoyi*. Transfer RNAs are designated by the IUPAC-IUB single letter amino acid codes (L1: trnL^CUN^; L2: trnL^UUR^;S1: trnL^AGN^; S2: trnL^UCN^). Labeling from the outside to inside circle: genes encoded on the heavy strand, genes encoded on the light strand, positive or negative AT skew[(A−T)/(A+T)], BLAST *H*. *intermedius*, *H*. *sajori* and *P*. *brachypterus* results, GC content (peaks out/inside the circle indicate values higher or lower than average GC content, respectively), GC skew [(G−C)/(G+C)], respectively.

The complete mitogenome sequence of *H*. *quoyi* was a 16,525 bp circular molecule. The mitogenome was typical of other Beloniformes fish mitogenomes, including 13 PCGs (*cox1-3*, *nad1-6*, *nad4L*, *atp6*, *atp8 and cytb*), 22 transfer RNA genes (one for each amino acid and two each for serine and leucine), 2 rRNA genes (12S rRNA and 16S rRNA) and two non-coding regions (the control region (CR) and O_L_) (Fig 1 and Table 2). Twenty-three genes were transcribed on the heavy strand (H-strand), whereas the other genes (*nad6* and eight tRNA genes (Asn, Gln, Ala, Cys, Tyr, Ser (UCN), Glu, and Pro)) were oriented on the light strand (L-strand). The organization and composition in the *H*. *quoyi* mtDNA was identical to most of Beloniformes fish sequenced to date[27, 28].

**Table 2:** Characteristic constituents of the mitochondrial genome of *H*. *quoyi*.

Feature	Strand*	Position	Spacer (+)/Overlap (–)	Start/Stop codon
tRNA-Phe (F)	H	1–69	0	
12S rRNA	H	69–1012	-1	
tRNA-Val (V)	H	1012–1085	-1	
16S rRNA	H	1085–2771	-1	
tRNA-Leu (L1)	H	2772–2845	0	
*nad1*	H	2846–3820	0	ATG/TAA
tRNA-Ile (I)	H	3824–3895	3	
tRNA-Gln (Q)	L	3894–3964	-2	
tRNA-Met (M)	H	3963–4034	-2	
*nad*2	H	4034–5080	-1	ATG/TAG
tRNA-Trp (W)	H	5079–5151	-2	
tRNA-Ala (A)	L	5153–5221	1	
tRNA-Asn (N)	L	5223–5295	1	
O_L_	L	5296–5333	0	
tRNA-Cys (C)	L	5334–5400	38	
tRNA-Tyr (Y)	L	5401–5471	0	
*cox*1	H	5473–7026	1	GTG/TAA
tRNA-Ser (S1)	L	7033–7101	6	
tRNA-Asp (D)	H	7107–7180	5	
*cox*2	H	7185–7875	4	ATG/T
tRNA-Lys (K)	H	7876–7950	0	
*atp* 8	H	7951–8118	0	ATG/TAA
*atp* 6	H	8108–8791	-11	ATG/TAA
*cox*3	H	8791–9576	-1	ATG/TAA
tRNA-Gly (G)	H	9576–9646	-1	
*nad*3	H	9647–9997	0	ATG/TAA
tRNA-Arg (R)	H	9995–10065	-3	
*nad*4l	H	10065–10361	-1	ATG/TAA
*nad*4	H	10355–11732	-7	ATG/T
tRNA-His (H)	H	11733–11801	0	
tRNA-Ser (S1)	H	11801–11870	-1	
tRNA-Leu (L1)	H	11873–11947	2	
*nad*5	H	11947–13785	-1	ATG/TAA
*nad*6	L	13782–14303	-4	ATG/TAA
tRNAGlu (E)	L	14304–14372	0	
*cytb*	H	14376–15516	3	ATG/T
tRNA-Thr (T)	H	15517–15589	0	
tRNA-Pro (P)	L	15590–15659	0	
Control region	H	15660–16525	0	

*H and L refer to the heavy and light strand, respectively.

### Skewness, overlapping, and intergenic spacer regions

The nucleotide composition of the *H*. *quoyi* mitogenome was slightly biased towards A and T, accounting for 56.80%. The overall base nucleotide composition of the H-strand was as follows: A = 4,838 (29.28%), T = 4,550 (27.53%), G = 2,534 (15.33%), and C = 4,603 (27.86%). The highest A+T content (65.24%) was detected in the CR, which was consistent with previous reports of the skewness of teleostean species. The average AT-skew of Beloniformes mtDNA was 0.0089±0.0269, ranging from 0.0425 in *Dermogenys pusilla* to −0.0517 in *Hyporhamphus intermedius*[9, 29]. The AT-skew in *H*. *quoyi* mitogenome was positive (0.0307), which was similar to most mitogenomes of Exocoetidae, Belonidae, Scomberesocidae and Zenarchopteridae (Table 1). Among all sequenced Beloniformes mitogenomes, *H*. *quoyi* has a the most negative GC-skew (−0.2980) indicating that a higher content of Cs compared to Gs. Similar GC-skew values were also detected in other Beloniformes mitogenomes, apart from *Ablennes hians*[2]. Additionally, the mitogenome had a 31 bp overlap between genes in eleven locations ranging from 1 to 11 bp. Two overlaps, *atp8-atp6*(11 bp) and *nad4l-nad4*(6 bp), were detected in the *H*. *quoyi* mitogenome. The same phenomenon occurred in the Metazoa[30, 31]. There was a 69-bp nucleotide sequence dispersed in twelve intergenic spacers, ranging in size from 1 to 38 bp, with the longest spacer sequence located between the trnN and the trnC, which formed the origin of the light strand.

### Transfer RNA genes and ribosomal RNA genes

**Fig 2:**
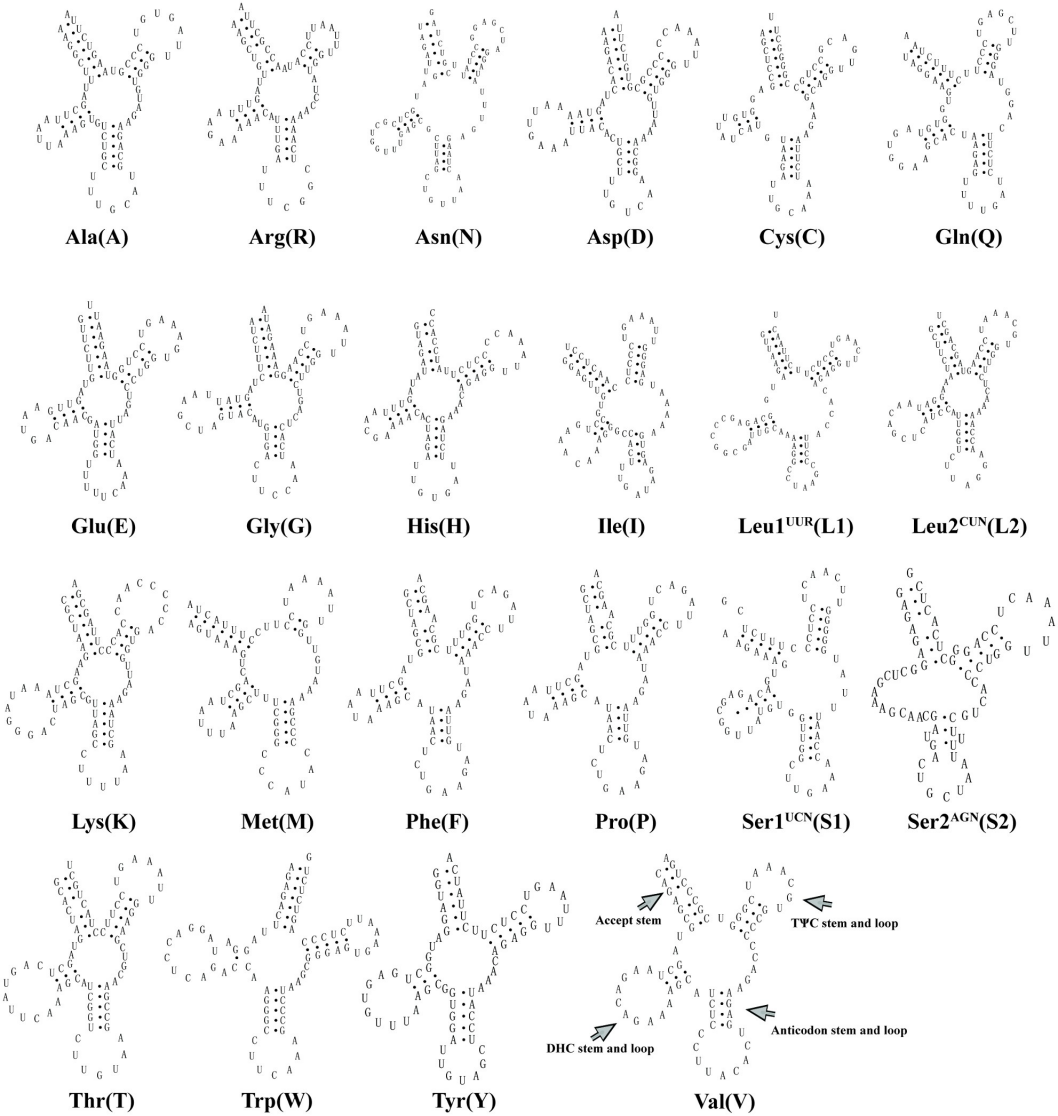
Secondary structures of transfer tRNAs in the *H*. *quoyi* mitogenome.

A total of 22 tRNA genes in the *H*. *quoyi* mitogenome were identified successfully based on their potential secondary structures (Fig 2). With the exception of 8 tRNAs, all other tRNAs were encoded by the H-strand (Table 2). The length of tRNAs of *H*. *quoyi* ranged from 66 bp to 74 bp in size. Most of the tRNA genes could be folded into typical cloverleaf secondary structures, except trnS2 (AUN) lacking of a DHU stem. This phenomenon occurs in most teleost mitogenomes including Beloniformes species [31–33]. Although almost all secondary structures of tRNAs had amino acid acceptor stem with 7 bp paired bases, the remaining trnaF, trnaV, trnaE and trnaP have a 9 bp aminoacyl acceptor stem. A total of 16 unmatched base pairs (G-U pairs) were found in the *H*. *quoyi* tRNAs, which form a weak bond. A positive AT skew (0.1209) and a negative GC skew (−0.1250) were found among the concatenated sequences of all 22 tRNAs in *H*. *quoyi*, indicating tRNAs biased toward As and Cs. Similar results had been found in the ribosomal genes. The AT skew of 12S and 16S rRNA genes were 0.1610 and 0.2425, respectively, and they had a negative GC skew (−0.0930 and −0.0992). The length of the 12S rRNA and 16S rRNA were 944 bp and 1,687 bp and A+T contents were 53.28% and 56.97%, respectively. The location of the 12 rRNAs was between trnF and trnV, and the location of the 16 rRNAs was between trnV and trnL1 (UUR), which were similar for most vertebrates [31, 34, 35].

### Protein-coding genes

**Fig 3:**
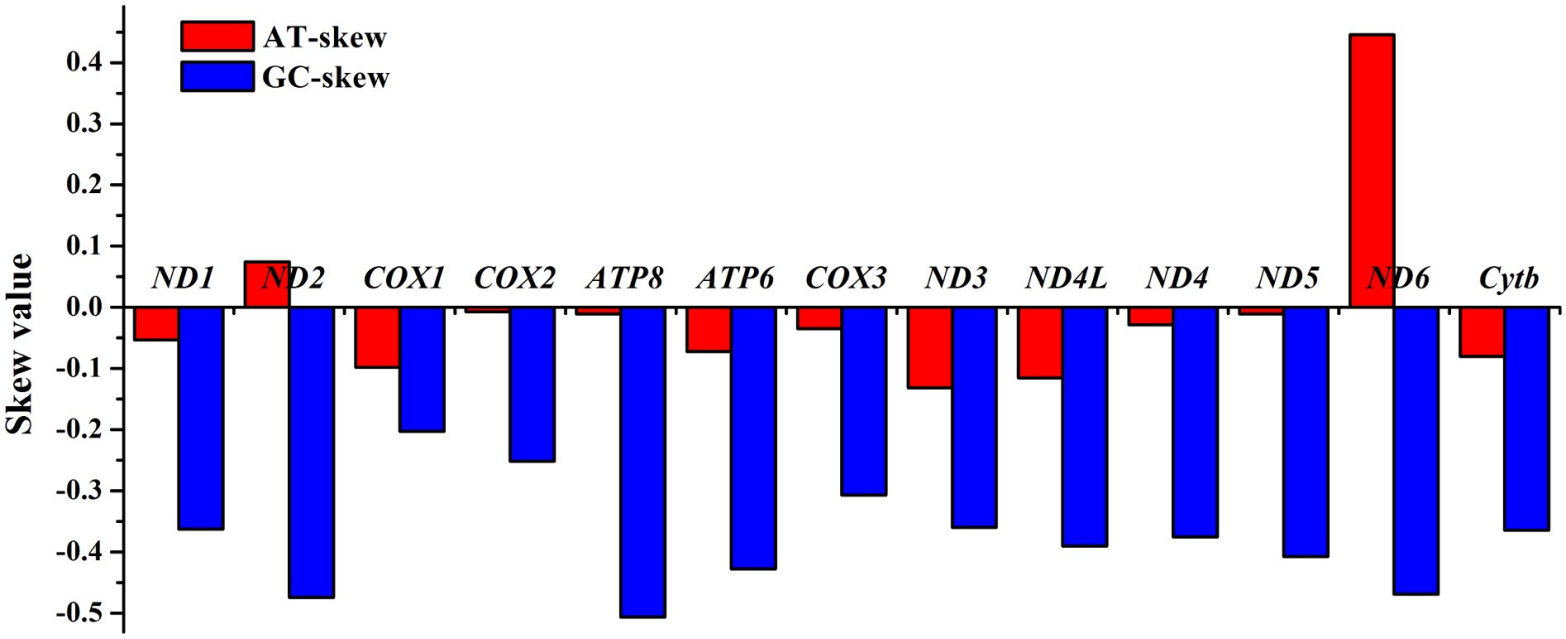
The AT and GC skew in the PCGs of the *H*. *quoyi* mitogenome.

The 13 PCGs in *H*. *quoyi* mitogenome comprised 11,433 bp in total, with a A+T content of 56.50%, and ranged in size from 168 bp (*atp6*) to 1,839 bp (*nad5*). The start and stop codons of the 13 PCGs in the *H*. *quoyi* mtDNA were shown in Table 2. All but one PCGs of *H*. *quoyi* initiated with methionine (ATG) as the start codon. The only exception was the *cox1* gene, which utilized GTG as a start codon. The phenomenon of alternative start codons occurs in most teleost mitogenomes[8, 31, 32]. The majority of the PCGs of *H*. *quoyi* had the complete termination codons TAA (*nad1*, *nad3*, *nad4l*, *nad5*, *nad6*, *cox1*, *cox3*, *atp6* and *atp8*) or TAG (*nad2*). The remaining three genes (*cox2*, *nad4* and *cytb*) utilized T as incomplete termination codons, which were presumed to be completed through post-transcriptional RNA editing mechanism in metazoan mitogenomes[36]. The AT skew and GC skew values of the PCGs were shown in Fig 3. All PCGs of GC skew and AT skew values were negative, except for *nad2* and *nad6*, indicating most PCGs contained more Ts and Cs, which was identical to most previous observations[6, 28, 35].

**Fig 4:**
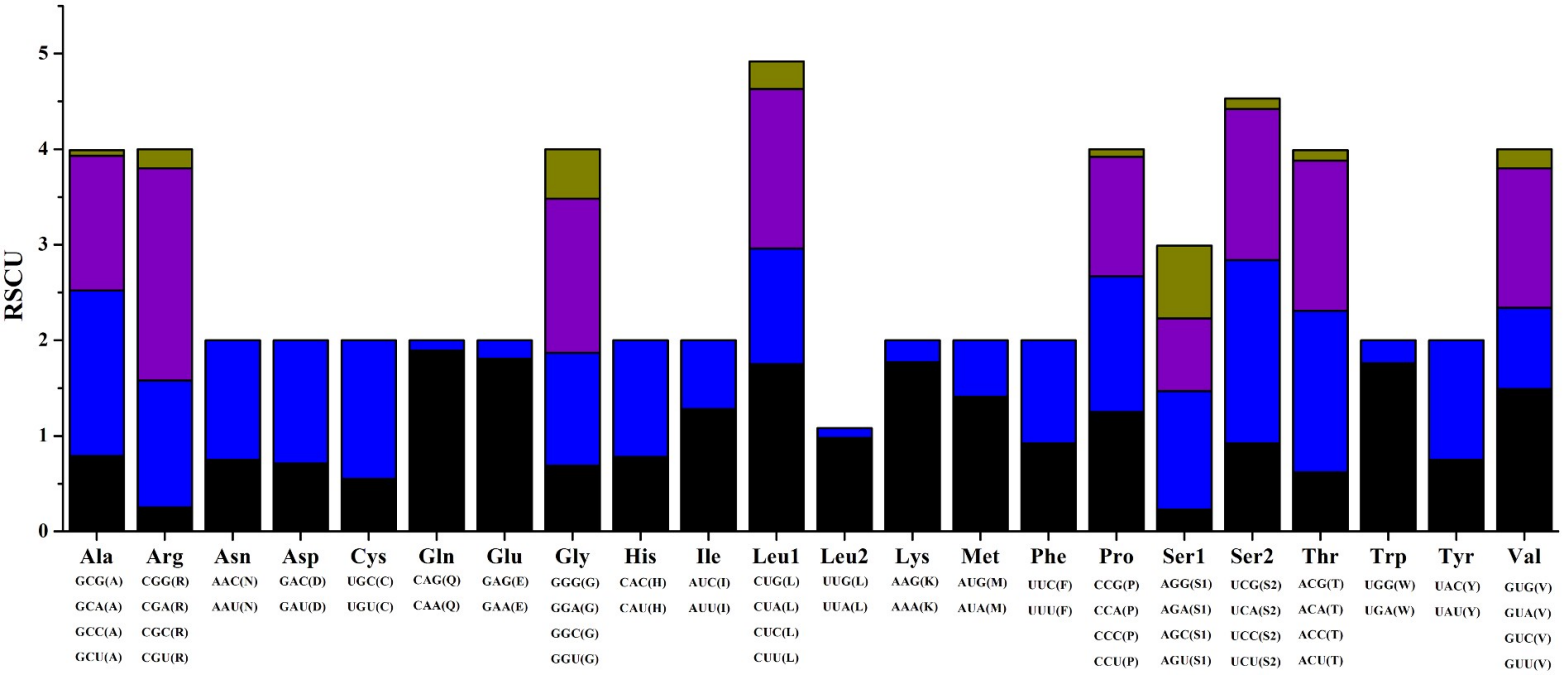
RSCU in the mitogenomes of *H*. *quoyi*.

**Fig 5:**
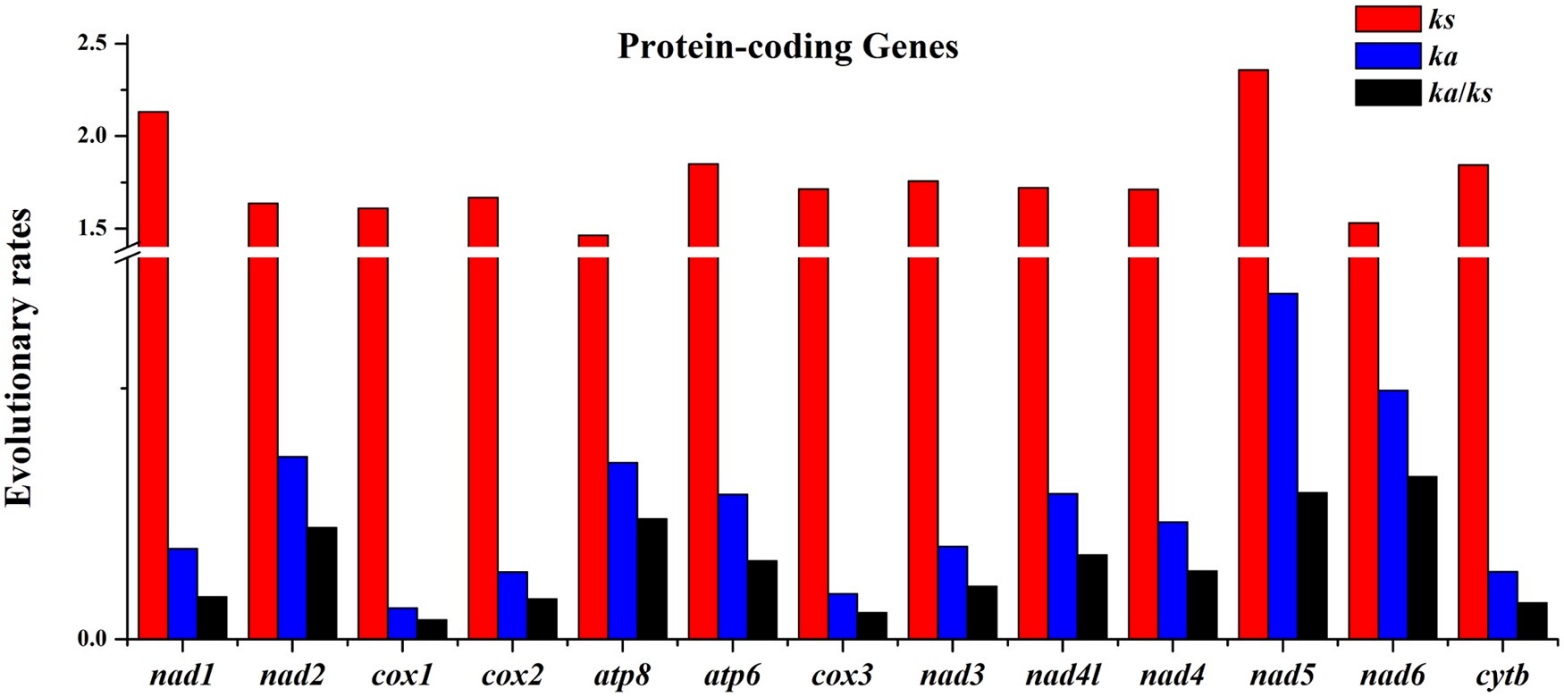
Evolutionary rates of *H*. *quoyi* mitogenome. Rate of non-synonymous substitutions (*Ka*), rate of synonymous substitutions (*Ks*) and ratio of the rate of non-synonymous substitution to the rate of synonymous substitution (*Ka*/*Ks*) for each PCGs are shown.

RSCU for the *H*. *quoyi* mtDNA were shown in S2 Table and Fig 4. The value greater than 1 mean the codon more commonly used. Nine amino acid were encoded by four different codons and 13 amino acid were encoded by two codons. Excluding AGA and AGG codons, the total number of codons in PCGs of *H*. *quoyi* was 3792. The most common amino acids were Leucine 1 (Leu 1, 552), alanine (Ala, 292)and threonine (Thr, 335) in *H*. *quoyi*. In all 13 PCGs, the *Ka*/*Ks* ratio was much less than 1 (varied from 0.0192 (*cox1*) to 0.1618 (*nad6*)) (Fig 5), indicating that all the PCGs were evolving under the purifying selection. The result suggested negative selective coefficients affected purifying selection against deleterious mutations [37]. In addition, the highest ratios were in *nad5* and *nad6* in the H- and L-strand, respectively, indicating that the selection pressures were relatively independent on the two strands.

### Non-coding regions

**Fig 6:**
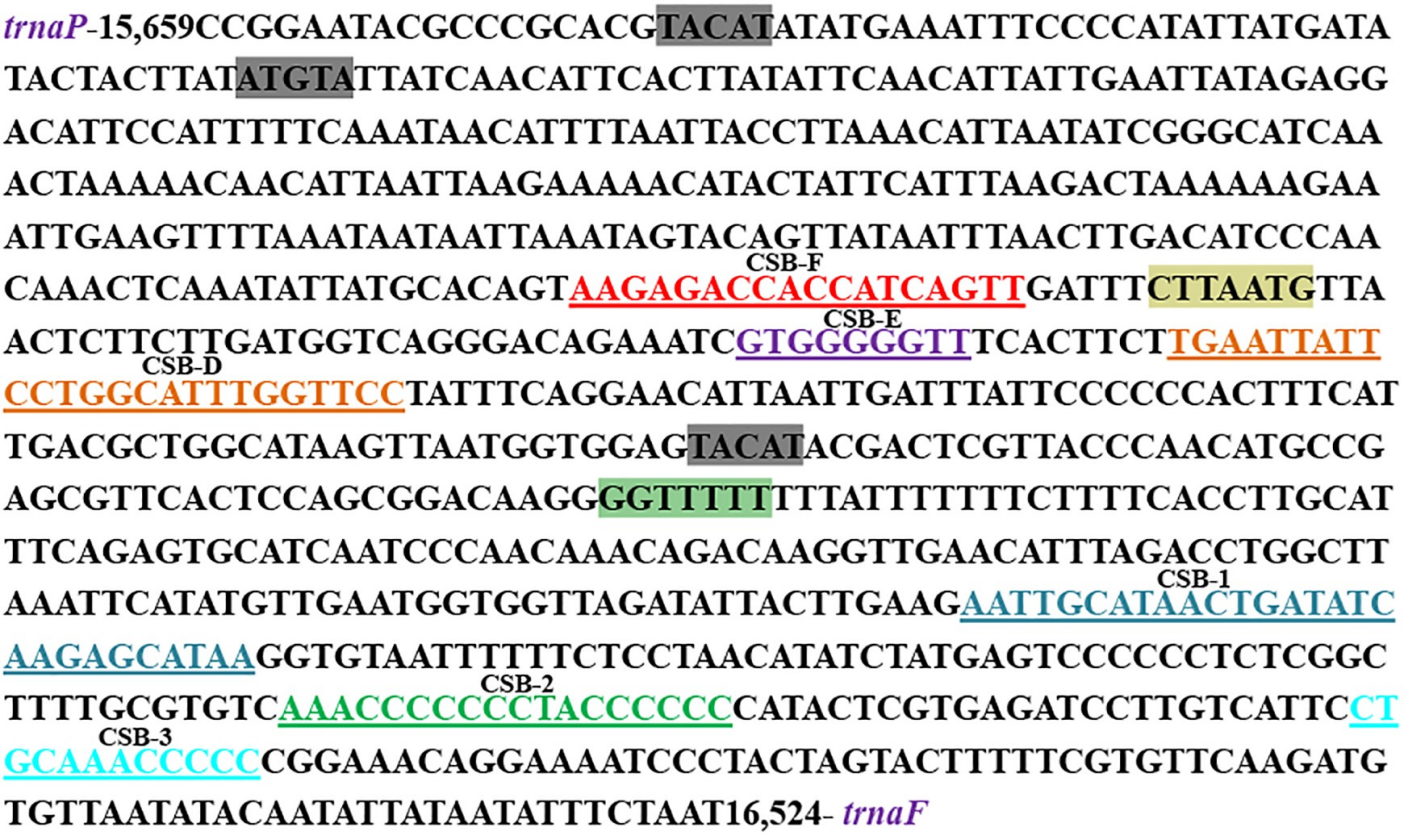
Features present in the control regions of the *H*. *quoyi* mitogenome. The gray background denote conserved motifs ATGTA and its complement TACAT. The relatively similar repetitive motifs (GGTTTTT) have green background and highly conserved motifs (CTTAATG) have yellow background.

The mtDNA had two long non-coding regions, O_L_ and CR, which were used for the replication, and maintenance of the mitogenome. A 38 bp O_L_, which was folded into a hairpin secondary structure, was located between trnN and trnC (S1 Fig)[38]. The 866 bp long CR was found between tRNA^Pro^ and tRNA^Phe^ with 65.24% A+T content, which was essential for the initiation of vertebrate mtDNA replication[9, 31, 34]. Several conserved sequence blocks (CSBs), which could be very important roles for mitochondrial metabolism, were found in the CR of teleost fish[39]. The central conserved blocks (CSB-F, CSB-E and CSB-D) were found in the CR of *H*. *quoyi*, and the conserved sequence block domains (CSB-1, CSB-2 and CSB-3) were similarly detected (Fig 6). By comparing the recognition sites in Beloniformes species, all of the CSBs were typically present in CR of teleost fish [19, 39]. The relatively similar repetitive motifs (GGTTTTT) and highly conserved motifs (CTTAATG) were found in CR of *H*. *quoyi*. Besides, tandem repeats were not recognized in *H*. *quoyi*. Beyond the genera *Oryzias* and *Ablennes*, tandem repeats did not similarly appeared in other Beloniformes fish [2, 32].

### Phylogenetic analysis

**Fig 7:**
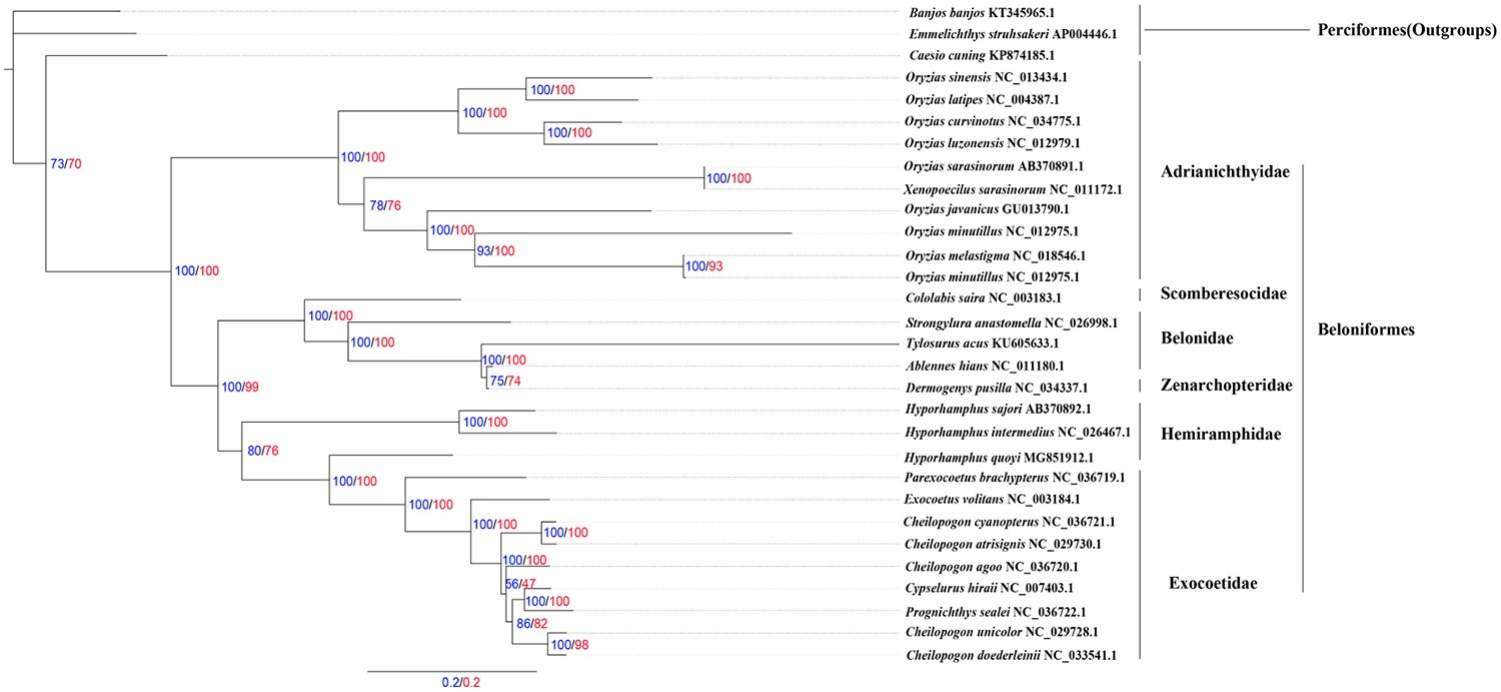
Inferred phylogenetic relationships among Beloniformes by the ML methods based on concatenated nucleotide and amino acid sequences of the 13 PCGs, Perciforme fish, *C*. *cuning* (KP874185.1), *E*. *struhsakeri* (AP004446.1) and *B*. *banjos* (KT345965.1) as outgroups. The numbers along branches indicate ML bootstrap values based on concatenated nucleotide (blue numbers) and amino acid (red numbers) sequences of the 13 PCGs, respectively.

**Fig 8:**
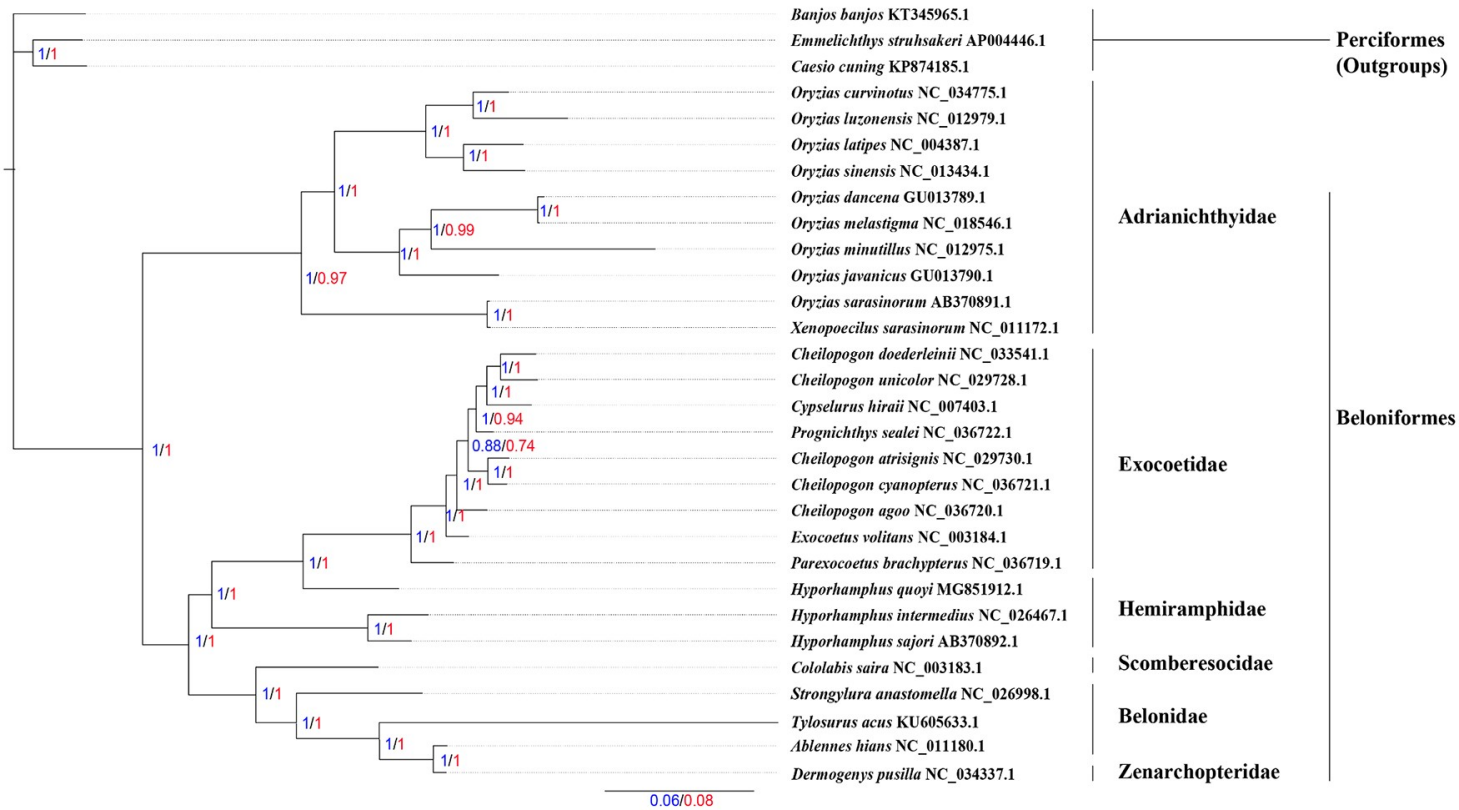
Inferred phylogenetic relationships among Beloniformes by the BI methods based on concatenated nucleotide and amino acid sequences of the 13 PCGs, using Perciforme fish, *C*. *cuning* (KP874185.1), *E*. *struhsakeri* (AP004446.1) and *B*. *banjos* (KT345965.1) as outgroups. The numbers along branches indicate Bayesian posterior probability values based on concatenated nucleotide (blue numbers) and amino acid (red numbers) sequences of the 13 PCGs, respectively.

The phylogenetic relationships of Beloniformes were constructed by the BI and ML methods based on concatenated nucleotide and amino acid sequences of the 13 PCGs from 27 Beloniformes species and three outgroups species (Figs 7 and 8). The phylogenetic trees contained consistently three major clades, including (I) Adrianichthyidae, (II) Scomberesocidae, Belonidae and Zenarchopteridae, (III) Hemiramphidae and Exocoetidae. The best supported phylogenetic relationship of Beloniformes is as follows: (Adrianichthyidae + ((Hemiramphidae + Exocoetidae) + (Scomberesocidae + (Belonidae + Zenarchopteridae))). We sequenced *H*. *quoyi* within Hemiramphidae as the sister group to Exocoetidae, and *H*. *quoyi* in comparison to the other two Hemiramphidae species shared a close ancestry with Exocoetidae. This result may be that the mitogenome of *H*. *quoyi* more close to *P*. *brachypterus* within Exocoetidae than the other two Hemiramphidae fish based on BLAST analysis, and especially in *nad2* (Fig 1). The topology relationships of Beloniformes was consistent with most phylogenetic mitogenomes research[1, 19]. However, previous work based on partial mitochondrial gene (16S and *cytb*) and nuclear genes (*Rag2* and *Tmo*) for phylogenetic analysis indicated that Hemiramphidae was close to Belonidae besides Exocoetidae[40]. Whether the difference in the phylogenetic analysis is due to e.g. hybridization, introgression and lineage sorting is unknown. It is worth noting that the phylogenetic placement of *H*. *quoyi* inferred here actually makes Hemiramphidae paraphyletic. Moreover, the previous research based on nuclear genes also showed that Hemiramphidae including *H*. *quoyi* and *H*. *sajori* was a paraphyletic grouping[40]. Besides, each of the family Zenarchopteridae and Scomberesocidae were only one mitogenome sequenced to date. Additional mitogenomes data from Zenarchopteridae, Scomberesocidae and Hemiramphidae fish are required to demonstrate the relationships among Beloniformes species in the future.

## Supporting information

S1 Table Primer pairs used for PCR amplification of *H*. *quoyi* mitogenome. (XLSX)

S2 Table Codon number and RSCU in *H*. *quoyi* mitochondrial PCGs. A total of 3,792 codons were analysed excluding the initiation and termination codons. Amino acids encoded by these codons are labelled according to the IUPAC-IUB single-letter amino acid codes.
(XLSX)

S1 Fig The putative hairpin secondary structure of the O_L_ of the *H*. *quoyi* mitogenome. (TIF)
