# Supplementary figures and images for: Mitochondrial genome of the garfish Hyporhamphus quoyi (Beloniformes: Hemiramphidae) and phylogenetic relationships within Beloniformes based on whole mitogenomes
Source: PLoS One. 2018 Nov 15;13(11):e0205025. doi: 10.1371/journal.pone.0205025 (PMC6237333; doi:10.1371/journal.pone.0205025)

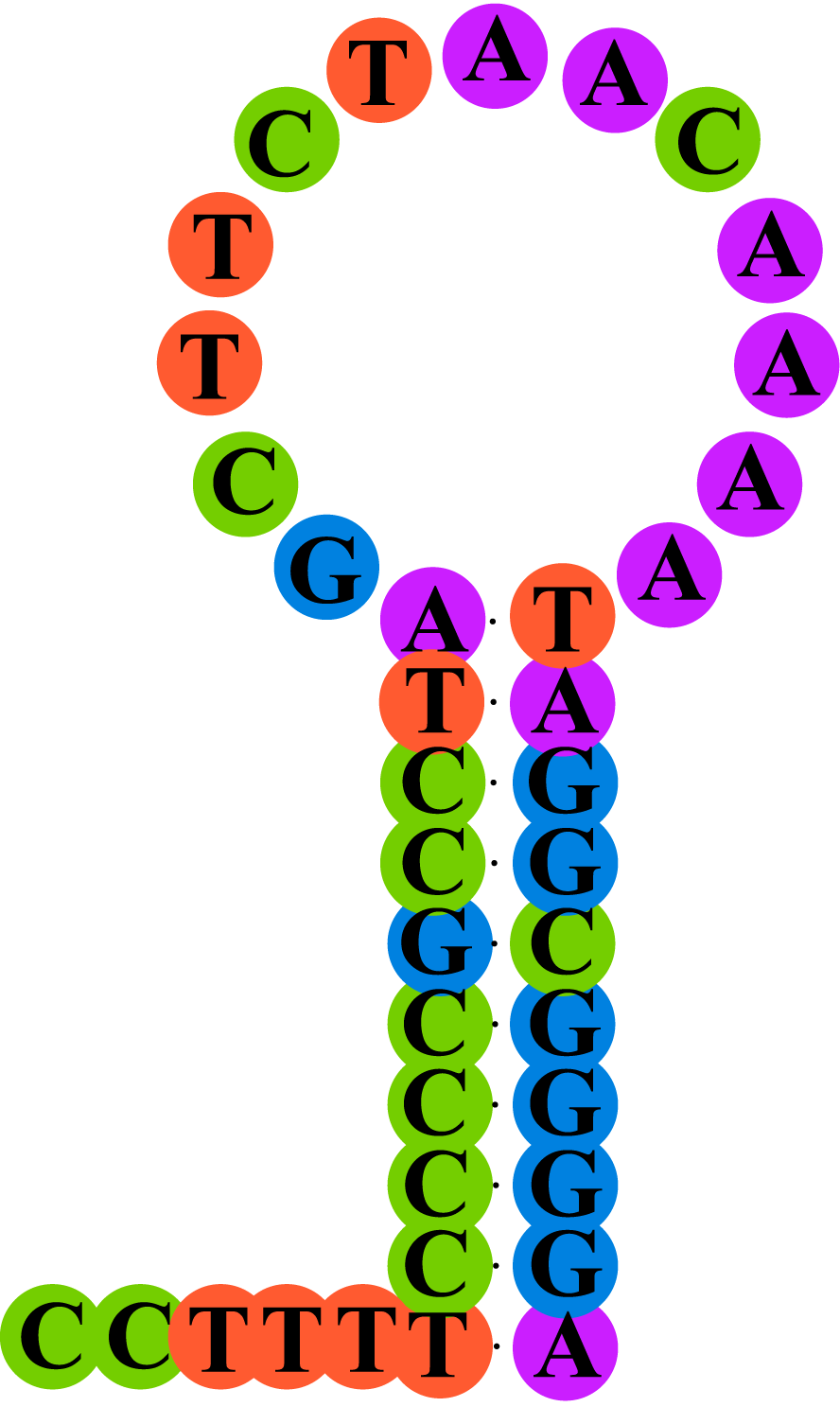

Supplement: S1 Fig — (TIF) [file pone.0205025.s003.tif]
